# Matrix Metalloproteinase-3 (MMP-3) Is an Endogenous Activator of the MMP-9 Secreted by Placental Leukocytes: Implication in Human Labor

**DOI:** 10.1371/journal.pone.0145366

**Published:** 2015-12-29

**Authors:** Arturo Flores-Pliego, Aurora Espejel-Nuñez, Marisol Castillo-Castrejon, Noemi Meraz-Cruz, Jorge Beltran-Montoya, Veronica Zaga-Clavellina, Sonia Nava-Salazar, Maribel Sanchez-Martinez, Felipe Vadillo-Ortega, Guadalupe Estrada-Gutierrez

**Affiliations:** 1 Instituto Nacional de Perinatologia Isidro Espinosa de los Reyes, Mexico City, Mexico; 2 Unidad de Vinculacion de la Facultad de Medicina, UNAM en el Instituto Nacional de Medicina Genomica, Mexico City, Mexico; Shanghai Jiaotong University School of Medicine, CHINA

## Abstract

**Background:**

The activity of matrix degrading enzymes plays a leading role in the rupture of the fetal membranes under normal and pathological human labor, and matrix metalloproteinase-9 (MMP-9) it is considered a biomarker of this event. To gain further insight into local MMP-9 origin and activation, in this study we analyzed the contribution of human placental leukocytes to MMP-9 secretion and explored the local mechanisms of the pro-enzyme activation.

**Methods:**

Placental blood leukocytes were obtained from women at term gestation without labor and maintained in culture up to 72 h. MMP-9 activity in the culture supernatants was determined by zymography and using a specific substrate. The presence of a potential pro-MMP-9 activator in the culture supernatants was monitored using a recombinant biotin-labeled human pro-MMP-9. To characterize the endogenous pro-MMP-9 activator, MMP-1, -3, -7 and -9 were measured by multiplex assay in the supernatants, and an inhibition assay of MMP-9 activation was performed using an anti-human MMP-3 and a specific MMP-3 inhibitor. Finally, production of MMP-9 and MMP-3 in placental leukocytes obtained from term pregnancies with and without labor was assessed by immunofluorescence.

**Results:**

Placental leukocytes spontaneously secreted pro-MMP-9 after 24 h of culture, increasing significantly at 48 h (*P*≤0.05), when the active form of MMP-9 was detected. Culture supernatants activated the recombinant pro-MMP-9 showing that placental leukocytes secrete the activator. A significant increase in MMP-3 secretion by placental leukocytes was observed since 48 h in culture (*P*≤0.05) and up to 72 h (*P*≤0.001), when concentration reached its maximum value. Specific activity of MMP-9 decreased significantly (*P*≤0.005) when an anti-MMP-3 antibody or a specific MMP-3 inhibitor were added to the culture media. Placental leukocytes from term labor produced more MMP-9 and MMP-3 compared to term non-labor cells.

**Conclusions:**

In this work we confirm that placental leukocytes from human term pregnancies are able to secrete large amounts of MMP-9, and that the production of the enzyme it is enhanced by labor. We also demonstrate for the first time that endogenous MMP-3 plays a major role in MMP-9 activation process. These findings support the contribution of placental leukocytes to create the collagenolytic microenvironment that induces the rupture of the fetal membranes during human labor.

## Introduction

Rupture of the fetal membranes is the last event in the expulsive phase of normal human labor [1] or the unique clinical feature in the premature rupture of the membranes (PROM). This phenomenon is the result of extracellular matrix (ECM) degradation in these tissues [2], and it is associated to increased mechanical forces generated by the contracting uterus [3].

Degradation of ECM in the fetal membranes is a complex process that requires a network of cell signaling as well as secretion and activation of different matrix metalloproteinases (MMPs) [4]. These biochemical changes must be strictly synchronized to myometrial contractions and cervical ripening for a successful labor. PROM is the clinical evidence that changes leading to rupture of the membranes may be activated independently of other labor events such as myometrial activation. Mechanisms of PROM involve abnormal secretion and activation of MMPs. Cellular sources of MMPs and the specific mechanisms of induction and activation of these enzymes under normal and pathological conditions are not well understood.

MMPs are a family of zinc and calcium-dependent neutral endopeptidases which are subdivided according to their structural and functional properties into five groups: collagenases, gelatinases, stromelysins, matrilysins, membrane type and a heterogeneous group [5]. All family members are synthesized and secreted to the extracellular space as inactive pro-enzymes with a propeptide region that must be removed to become active. The biochemical mechanism that initiate the catalytic activity of MMPs is fairly known but seems to occur through four different mechanisms: (i) the extracellular activation of MMPs by non-MMP proteins [6], (ii) activation by other MMPs [7]; (iii) the intracellular activation of MT1-MMP (MMP-14) and MMP-11 by furin [8]; and (iv) MT-MMP-associated activation of MMP-2.

A few MMPs, including collagenase-1 (MMP-1) [9], gelatinase-A (MMP-2), gelatinase-B (MMP-9), stromelysin-1 (MMP-3) [10], matrilysin (MMP-7) [11], neutrophil collagenase (MMP-8) [12] and collagenase 3 (MMP-13) [13] are known to be expressed during labor in humans and rats. Although their specific role in the degradation of the fetal membranes ECM has not been completely characterized, a major role in this process has been ascribed to MMP-9 whose selective expression makes it a molecular marker of the onset of human labor [14, 15].

Preliminary studies aimed to identify the specific source of MMP-9 within gestational tissues have shown that leukocytes circulating in the placental blood produce several mediators that promote collagenolysis in the fetal membranes, resulting in, at least, an enhanced secretion and activation of MMP-9 during labor [16, 17]. The proenzyme form of MMP-9 must be activated to exert its proteolytic function, but the physiological mechanisms that control this activation during labor are unknown. In this context, the aim of this work was to analyze the specific contribution of placental leukocytes to MMP-9 secretion and explore into the endogenous mechanisms of the pro-enzyme activation to better understand the collagenolytic microenvironment during labor.

## Materials and Methods

### Ethics statement

The IRB of the Instituto Nacional de Perinatologia in Mexico City approved this study. Approval number 212250–02081. All participants signed the informed consent.

### Clinical samples

Placental samples were obtained under aseptic conditions from women who delivered by cesarean section at term (37–40 weeks of gestation), with intact membranes and no clinical evidence of labor or intrauterine infection. All procedures described underneath were carried out within the first hour of sample collection. For immunofluorescence assays, placentas from term labor women were used.

### Microbiological test

Placental swabs were rolled onto Columbia agar with 5% sheep blood, which was used as a primary isolation medium for fastidious and non-fastidious aerobic microorganisms. Appropriate selective media for detection of specific pathogens were used to discard infection. An additional swab was inoculated into Urea-Arginine LYO2 broth (bioMérieux) to detect infection due to mycoplasma species.

### Isolation of intervillous placental blood leukocytes

Once placenta was delivered, intervillous blood was drained out by manually compressing the cotyledons and recovered in sterile tubes containing heparin as anticoagulant (Becton-Dickinson, Rutheford, NJ). Leukocytes were isolated from placental blood by density gradient using Lymphoprep (Axis-Shield, Oslo, Norway) and cell viability over 95% was confirmed by staining with trypan blue. Cells were cultured in Dulbecco’s Modified Eagle Medium, supplemented with 0.2% lactalbumin hidrolysate (HLA), 1% sodium piruvate and 1% antibiotic-antimycotic (DMEM/HLA; Gibco BRL, Grand Island NY, USA). Leukocytes (5x10^5^ cells) were incubated in 12-well plates (Corning Costar, NY, USA) with 700 μl of DMEM/HLA for 24, 48, and 72 h at 37°C and 5% CO_2_. Supernatants were recovered by centrifugation at 1200 rpm for 15 min and stored to -70°C until use. Cell viability and functionality at each experimental time was assessed using the XTT-reduction method and IL-1b secretion by ELISA (R&D Systems, Minneapolis, MN), respectively.

### Gelatin zymography for MMP-9

Gelatinase activity was determined in placental leukocyte supernatants by SDS-PAGE containing 8% polyacrylamide and 1% gelatin under non-denaturing conditions as previously described [18]. Culture medium (0.5 μg protein) was loaded into each well. Supernatant of U-937 promyelocytic cell line was included as MMP-9 enzymatic activity standard [19]. The qualitative activity of MMP-9 in the supernatants was determined by image analysis (UVP, Cambridge, UK) and expressed as arbitrary optical density units (n = 7).

### MMP-9 enzymatic activity

Gelatinase activity in placental leukocyte supernatants was evaluated using a specific substrate (Ac-Pro-Leu-Gly-S-Leu-Leu-Gly-OC2H5, Calbiochem, San Diego, CA), according to Weingarten and Feder [20]. Supernatants (1.0 μg protein) in a solution containing 0.3 mM substrate, 50 mM HEPES (pH 7.4), and 1.0 mM 4,4′-dithiodipyridine (both from Sigma-Aldrich, St. Louis, MO), were incubated in triplicates for 3 h at 25°C. The 4,4′-dithiodipyridine reacts with the mercaptan hydrolysis fragment to form products that were measured at 324 nm, using a DU800 spectrophotometer (Beckman Coulter, Fullerton, CA).

### Activation of pro-MMP-9

To identify the presence of a potential activator of pro-MMP-9 in the leukocyte supernatants, media collected at 24, 48 and 72 h (2.0 μg protein) were incubated for 2 h at 37°C with 0.2 μg recombinant human pro-MMP-9 (Calbiochem, San Diego, CA), previously labeled with biotin-NHS (Vector Laboratories, England). After incubation, supernatants were subjected to SDS-PAGE and proteins were transferred to an Inmobilon-P nitrocellulose membrane (Millipore, Medford, MA, USA), using the western blot protocol described by Towbin [21]. Identification of resulting MMP-9 species was made directly using a sensitive avidin/biotin-based peroxidase system (VectaStain ABC Universal kit, Vector, Burlingame, CA), and peroxidase activity was revealed by chemiluminescence (ECL, Amersham Pharmacia Biotech, USA).

### Multiplex assay for matrix metalloproteinase quantification

MMP-1, MMP-3, MMP-7 and MMP-9 concentration in the leukocyte supernatants was measured simultaneously using the Fluorokine MultiAnalyte Profiling (X-MAP) kit from R&D Systems (Minneapolis, MN) and a Bio-Plex system (Luminex for BioRad), according to the manufacturer's protocol. Luminex Data Collector software version 1.7 (Luminex Corporation, Austin, TX), and Bio-Plex Manager data reduction software (Bio-Rad Laboratories, Hercules, CA), were used for data analysis. Once standard curves were generated, MMP concentrations were interpolated for each sample using a 5-parameter curve fit equation.

### Inhibition of MMP-9 activation through inhibition of MMP-3

To characterize the activator of pro-MMP-9 in the culture media, a monoclonal anti-human MMP-3 antibody (Affinity BioReagent, Golden, CO) was added at the beginning of each culture (0 h) using a final concentration of 2.5 μg/mL. In addition, placental leukocyte cultures with 130 nM of a specific MMP-3 inhibitor (MMP-3 inhibitor II, Calbiochem, San Diego, CA) were running in parallel. After 24, 48, 72, and 96 h media were collected and analyzed for MMP-9 activity by gelatin zymography using the specific substrate, as previously described.

### Immunofluorescence for MMP-9 and MMP-3 in placental leukocytes

Placental leukocytes from term labor and term non-labor women were obtained and cultured as describe above. After 48 h, 15 μl of leukocytes in suspension were placed on silanized slides (Dako, Denmark) and fixed with 1:1 acetone/methanol. Cells were then treated with 1X PBS for 1 min and 1X PBS-0.2% Triton X100 for 5 min for permeabilization. Leukocytes were stained using a set of primary antibodies against MMP-9 and MMP-3 (Oncogene, La Jolla, CA) (dilution 1:50), and a FITC-conjugated secondary antibody (dilution 1:25) (Zymed Laboratories, San Francisco, CA). Finally, leukocytes were washed using a PBS-Evans Blue solution (5 drops in 100 ml of PBS) to contrast, mounted using Vectashield (Vector, Burlingame, CA), and observed under epifluorescent microscopy.

### Statistical analysis

Data were analyzed using the SPSS v.20 statistics program. Data analysis was performed with two-tailed ANOVA test to compare each group. Statistical significance was set at the 95% level (*P*≤0.05) and results were expressed as mean ± SD.

## Results

### Viability and functionality of placental leukocytes

**Fig 1:**
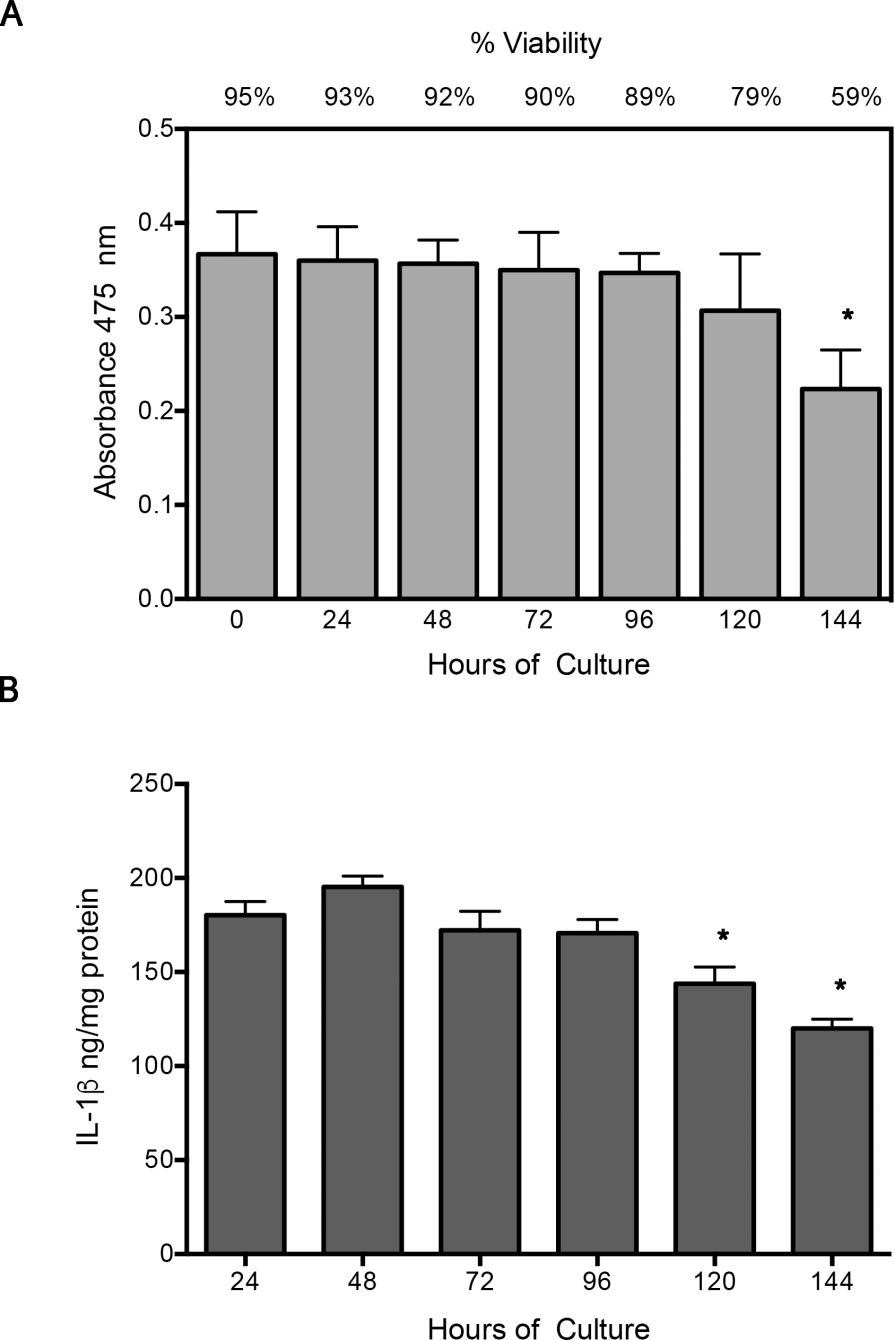
Placental leukocytes remain viable and functional during culture. A) XTT viability assay, time 0 was consider as 95% of viability according to trypan blue staining. B) IL-1b ELISA in the supernatants of placental leukocytes along the culture used as functionality assay. Placental leukocytes in culture remain viable (around 90%) and secreting IL-1b from 24 to 96 h, which was the culture period for all the experiments. Three independent experiments in duplicate are expressed as mean ± SD. *P≤0.05 compared to time 0 (A) or 24 h (B).

XTT assay shows that placental leukocytes in culture remained viable during experimental time (95–89%), with no statistical significant difference; only after 144 h of culture, viability decreased significantly (*P*≤0.05) compared to time 0, but cells were not used at this time. IL-1b secretion, as functional marker, remained constant during experimental time (24–96 h), declined significantly just until 120–144 h (*P*≤0.05) (Fig 1).

### Placental leukocytes secrete proenzyme and active MMP-9

**Fig 2:**
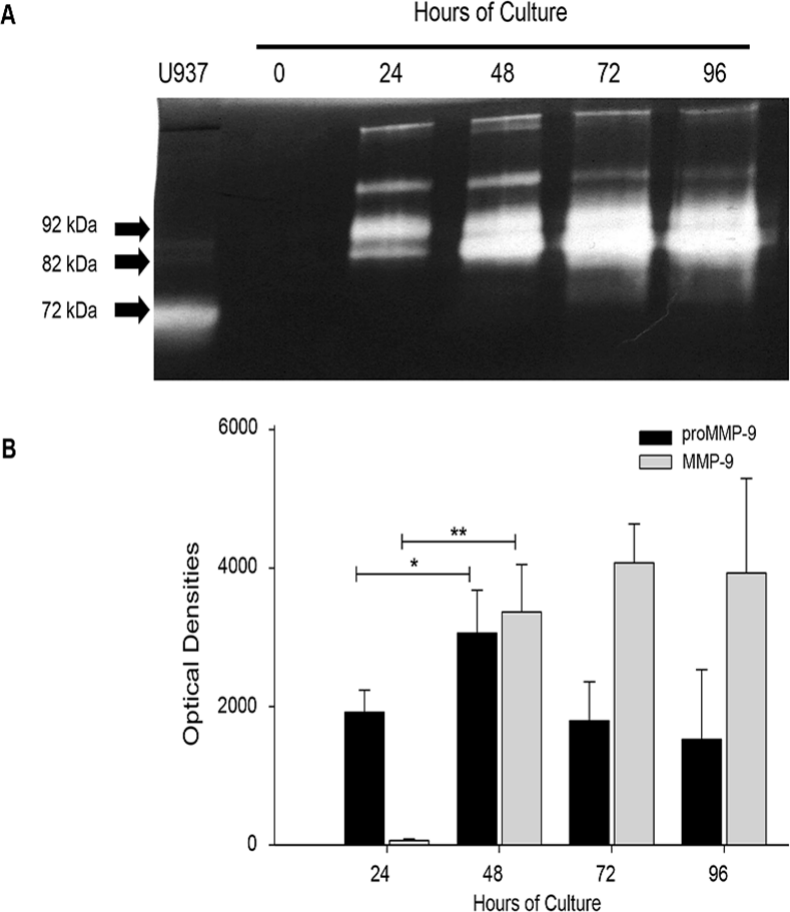
Placental leukocytes in culture secrete pro-enzyme and active MMP-9. A) Representative gelatin zymography (0.5 μg protein per lane) of placental leukocyte supernatants shows the 92 kDa pro-enzyme and the 82 kDa active form of MMP-9. B) The bars represent the optical densities (OD) of pro-enzyme and active MMP-9. While the pro-enzyme increased after 24h in supernatants, the active form was increased significantly only after 48h of culture. Seven independent experiments in duplicate are expressed as mean ± SD. *P≤0.05, **P≤0.005

Gelatin zymography showed that placental leukocytes secrete proMMP-9 (92 kDa) at 24 h of culture (1917 ± 318 optical densities OD), and significantly increased at 48 h (3059 ± 621 OD) (*P*≤0.05). The active form of MMP-9 was negligible at 24 h (52 ± 5 OD), but significantly increased after 48 h of incubation (3366.03 ± 685 OD) (*P*≤0.005), and was maintained throughout 96 h of culture. The presence of two additional lysis bands corresponding to gelatinases of higher molecular weight were also detected through the culture, and it is important to note that MMP-2 (72 kDa, another gelatinase, was not detectable in placental leukocyte supernatants (Fig 2).

**Fig 3:**
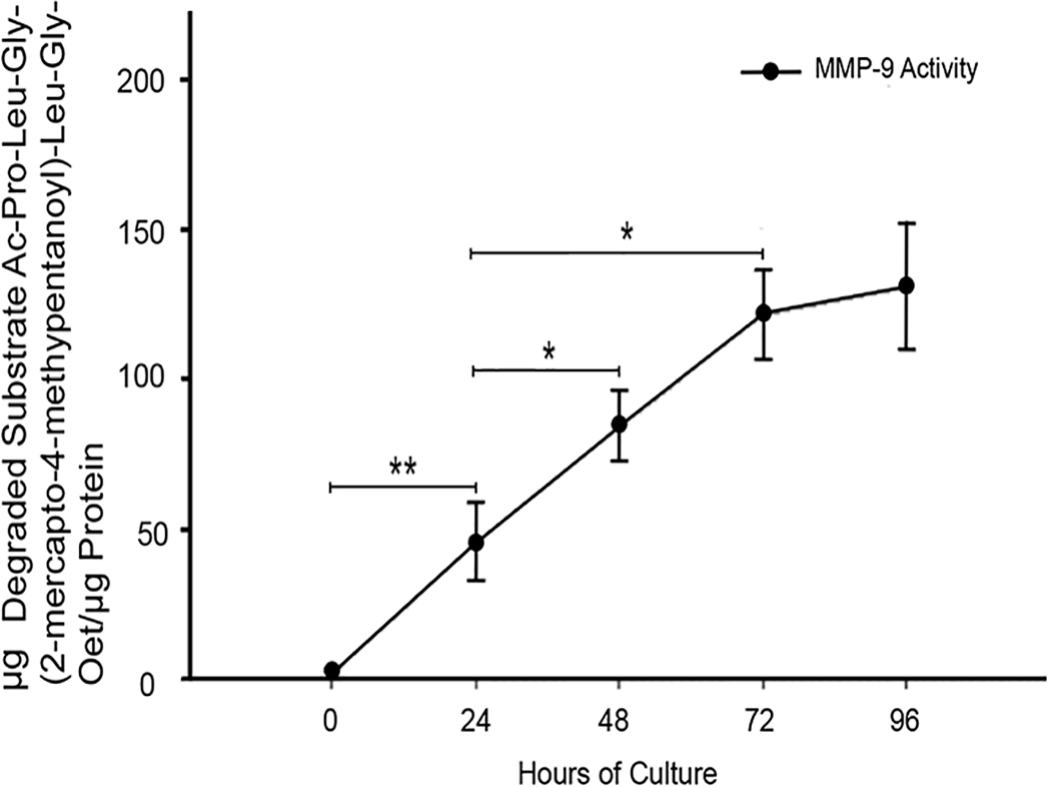
MMP-9 activity increases in placental leukocyte supernatants in a time-dependent manner. Specific substrate assay showed a significant increase in MMP-9 activity in the placental leukocyte supernatants along the culture, reaching the major activity at 72h. Each point represents the mean ± SD of 3 independent experiments in duplicate. *P≤0.05, **P≤0.005

These findings were confirmed with a specific MMP-9 activity assay (Fig 3), where a gradual increase in MMP-9 specific activity was observed, being significantly higher from 24 h (*P*≤0.005) until 72 h (45.53 ± 12.9 *vs* 121.6 ± 20.8 μg of degraded substrate per μg of protein, (*P*≤0.05) when exerts its major activity.

### Placental leukocytes produce an activator of proMMP-9

**Fig 4:**
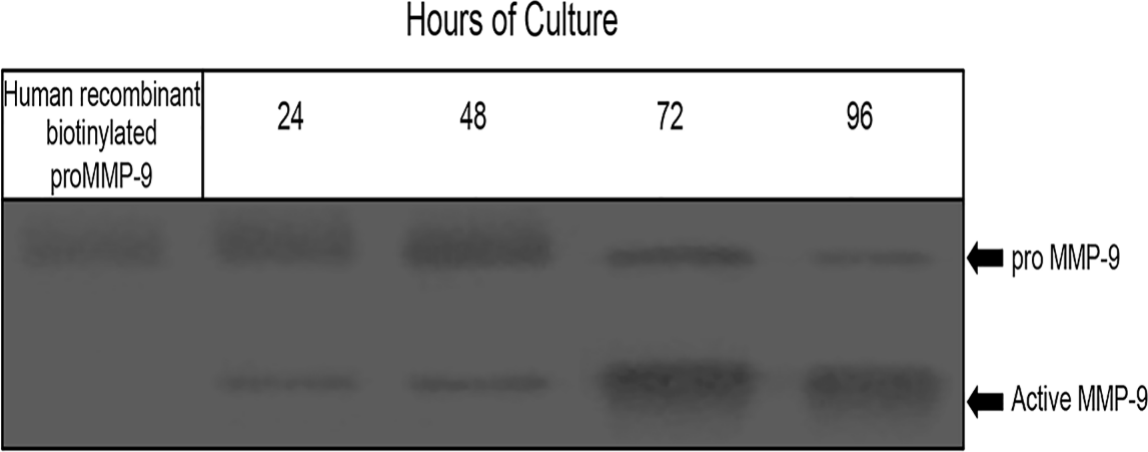
A natural activator of proMMP-9 is present in supernatants of placental leukocyte cultures. Representative western blot showing increasing activation of the exogenous recombinant proMMP-9 incubated in placental leukocyte supernatants. Three different supernatants were run in duplicate.

After the presence of both, proenzyme and active form of MMP-9 was confirmed, it was evaluated whether an activator of proMMP-9 was also present in the placental leukocyte supernatants. When biotinylated recombinant proMMP-9 was added to the culture media, exogenous enzyme became increasingly activated through the culture time, as demonstrated by western blot (Fig 4).

### Preliminar identification of the proMMP-9 activator

**Fig 5:**
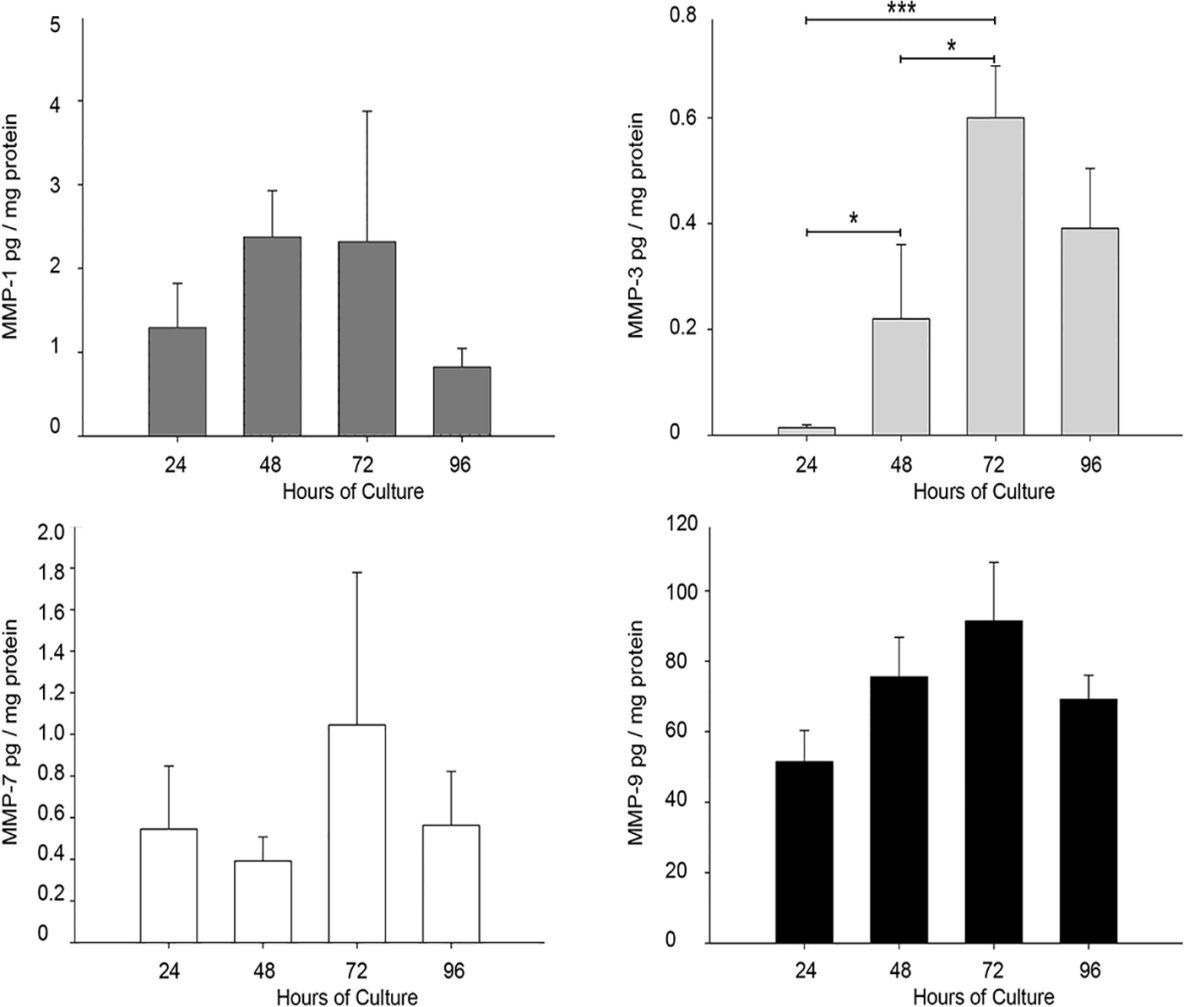
MMP-3 increments in placental leukocyte supernatants along the culture. Multiplex assay was performed in placental leukocyte supernatants to measure MMP-1, MMP-3, MMP-7 and MMP-9. MMP-3 showed a significant increase at 48 and 72h, while the other MMP-s did not significantly change. Bars represent mean ± SD of five independent experiments in duplicate. *P≤0.05, ***P≤0.001

As has been reported that MMP-1, 3, and 7 are potential activators of MMP-9 [22], a multiplex assay was conducted to measure the concentration of these four enzymes in the supernatants of placental leukocytes. Results showed no statistical differences in MMP-1, MMP-7 and MMP-9 concentration throughout the culture time, but a significant increase in MMP-3 secretion by placental leukocytes since 48 h of culture compared to 24 h (0.22 *vs* 0.0142 ± 0.0054 pg/mg protein; *P*≤0.05), and up to 72 h (0.60 ± 0.09 pg/mg protein; *P*≤0.001 compared to 24 h and *P*≤0.05 compared to 48 h), when concentration reached its maximum value (Fig 5).

### MMP-3 activates proMMP-9 in placental leukocytes

**Fig 6:**
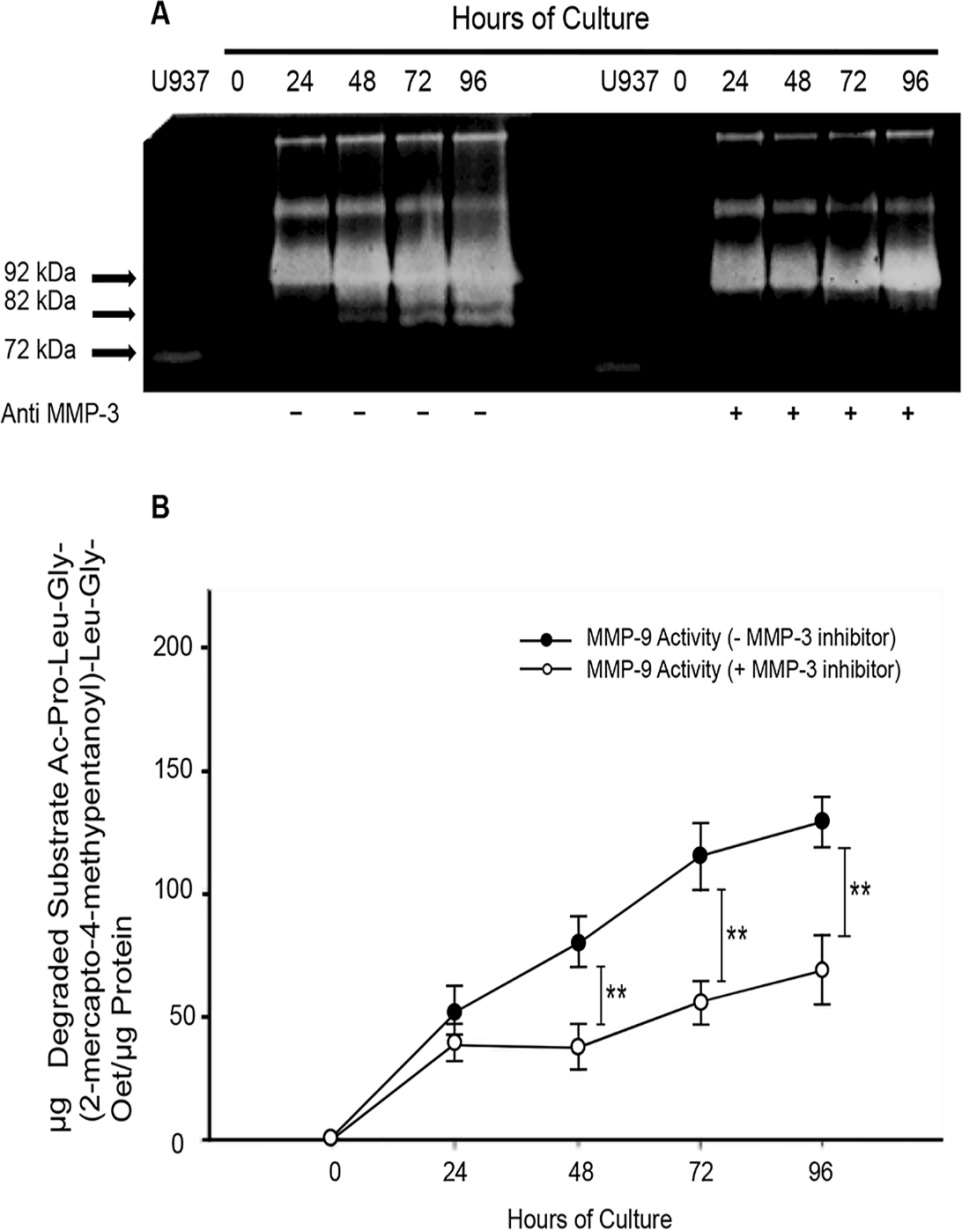
MMP-9 activation is inhibited by MMP-3. Cultures of placental leukocytes were incubated in the presence or absence of a neutralizing anti MMP-3 or a specific MMP-3 inhibitor. Culture media analyzed by gelatin zymography shows that the 82 kDa active form of MMP-9 disappears in the presence of the anti MMP-3 antibody at 24, 48 and 72 h (panel A representative gel, n = 3). MMP-9 activation is abolished by the specific MMP-3 inhibitor; each point represents the mean ± SD (**P<0.005) (panel B, n = 3).

As demonstrated before by gelatin zymography, the active form of MMP-9 (82 kDa) appears in placental leukocyte supernatants at 48 h of culture. However, when a recombinant anti MMP-3 was added to the placental leukocyte culture, the detection of the active MMP-9 was delayed until 72 h (Fig 6A). Accordingly, when a specific inhibitor of MMP-3 was added to the placental leukocyte culture, the specific activity of MMP-9 significantly decreased since 48 h (79.6498 ± 10.3919 *vs* 37.24 ± 9.93 μg of degraded substrate/μg protein; *P*<0.005); this significant low activity was maintained up to 96 h of culture (Fig 6B).

### Labor induces production of MMP-9 and MMP-3 by placental leukocytes

**Fig 7:**
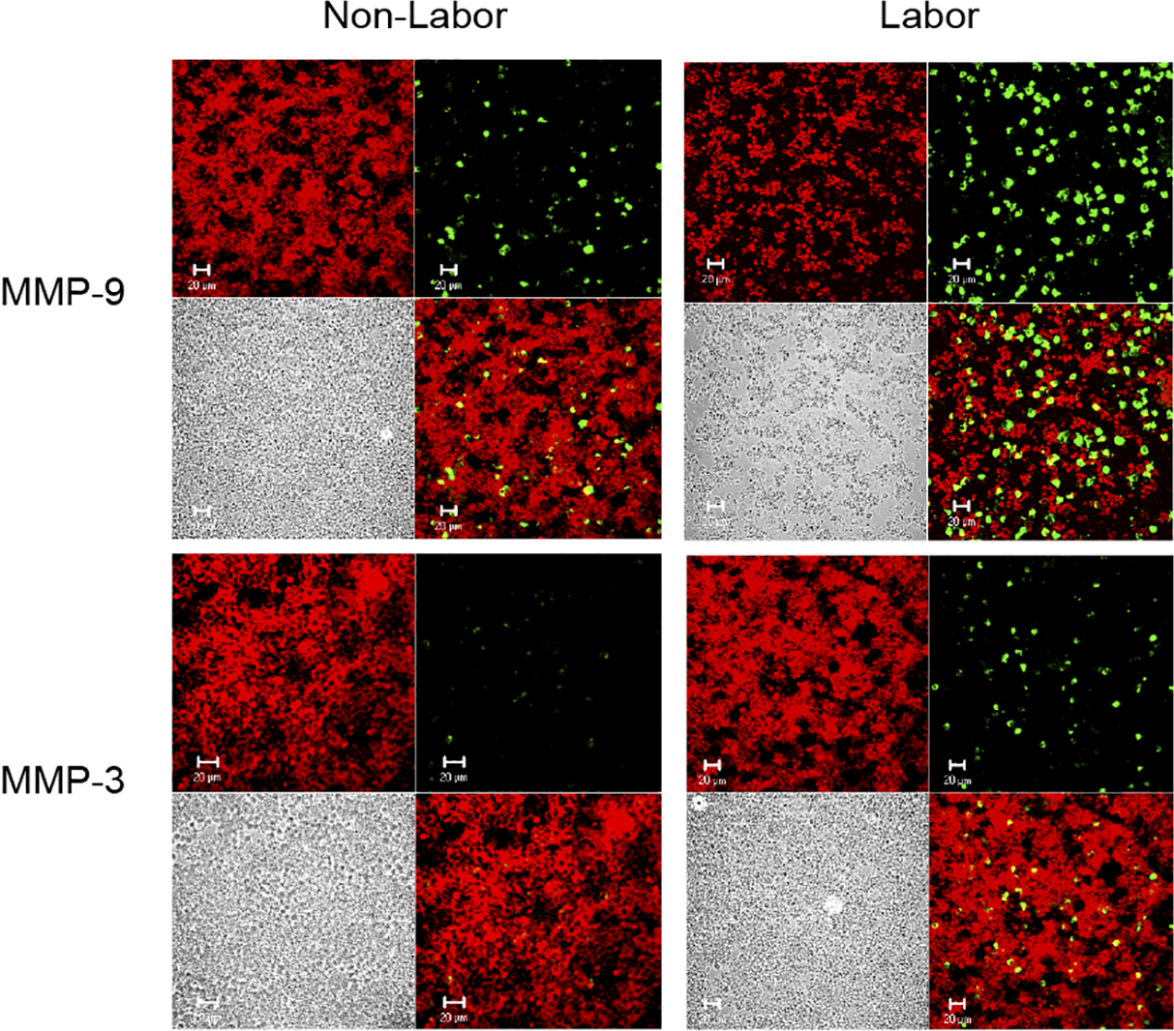
MMP-9 and MMP-3 are produced in large amounts during labor. Representative immunofluorescence images showing MMP-9 or MMP-3 immunolocalization in placental leukocytes obtained from term labor and non-labor women after 48 h of culture. Each panel shows Evans blue contrast staining (red) (upper left), MMP-9 or MMP-3 (green) (upper right), Nomarski interference contrast (lower left), and merge (lower right). Placental leukocytes from term labor produce more MMP-9 and MMP-3 compared to term non-labor cells. Confocal microscopy 40X.

In order to evaluate if the production of MMP-9 and MMP-3 by placental leukocytes it is influenced by labor, both enzymes were immunolocalized in placental leukocytes obtained from term pregnancies with or without labor. As shown in Fig 7, immunofluorescence revealed that placental leukocytes from women with labor produce more MMP-9 and MMP-3 than non-labor samples, as well as that MMP-9 is more abundant that MMP-3.

## Discussion

Accumulating evidence points to the arrival of different types of leukocytes to gestational tissues in the third trimester of pregnancy, which invade the fetal membranes and choriodecidua just before labor [23–25]. Upon homing in the intrauterine environment, these leukocytes produce different soluble mediators involved in the degradation of the connective tissue of the fetal membranes, as we have demonstrated before [15, 24]. These mediators include both, signaling molecules such as TNF-a and IL-1b, and MMPs, suggesting that leukocytes participate in the local network that regulate a highly compartmented collagenolytic microenvironment resulting in at least, weakening of the fetal membranes and cervical ripening [23, 26].

Several studies conducted by our research group and others have identified MMP-9 as a key player in the rupture of the fetal membranes [2, 16, 17, 27]. Therefore, in this work we further studied the role of placental leukocytes in MMP-9 secretion and explored into the mechanisms of pro-MMP-9 activation. Our results confirm that these leukocytes obtained from term pregnancies secrete pro-MMP-9 in culture [28].

When studying enzymatic activation, it is important to distinguish real activation of the pro-enzyme from its mere conversion into a lower molecular weight form that is catalytically inactive [29], therefore, we measured the activity of the 82 kDa form of MMP-9 using different approaches. Our results using a specific substrate confirmed that MMP-9 secreted by placental leukocytes is indeed catalytically active. Additionally, we were able to document gelatinolytic activity in bands of higher molecular weights, 115 and 215 kDa approximately. These enzymatic species may correspond to previously described associations of MMP-9 with other proteins such as lipocalin [neutrophil gelatinase-associated lipocalin (NGAL)], to produce a 125 kDa form, or a 225 kDa dimeric form [30, 31], which may be related to the ability of these cells to secrete various types of MMPs that facilitate the rupture of fetal membranes.

In this work, results show that under culture conditions, placental leukocytes activate MMP-9 to 82 kDa species after 48 h of culture. MMP-9 activity increased progressively along the first 72 h, meaning that placental leukocytes are able to maintain both secretion and activation mechanisms for at least 3 days. These findings suggest that placental leukocytes may also secrete the molecular machinery required for activating this enzyme.

Using the same leukocyte culture model, we next investigated a potential activator of pro-MMP-9, also secreted by these cells [32, 33]. By adding a biotin-labeled recombinant to the culture media, we were able to document the endogenous activation process by revealing the generation of novel 82 kDa active forms of MMP-9, previously observed. The 82 kDa labeled protein was observed in the culture media after 48 h, consistent with the results obtained by zymography. This result further confirms the presence of the activator of pro-MMP-9 in the culture media, and that the activation process is related to the expression of activators.

Several *in vitro* studies have documented the activation of pro-MMP-9 by other members of the MMPs family. In this context, MMP-1, MMP-3, and MMP-7, [34] have been proposed to constitute the main axis in the activation of pro-MMP-9 in addition to MMP-9 itself [22]. Therefore, we quantified these three MMPs in the culture media of placental leukocytes and only MMP-3 was found to increase significantly at 48 h of culture, coinciding with the emergence of the 82 kDa active form of MMP-9.

The addition of anti-MMP-3 neutralizing antibody to the culture greatly diminished the amount of the 82 kDa form of MMP-9 observed by zymography, suggesting that MMP-3 has indeed a major implication on the activation of pro-MMP-9 in our model. These findings were confirmed by using a specific MMP-3 inhibitor, which significantly decreased the specific activity of MMP-9 from 48 h until 96 h of culture. This activity, however, did not completely disappear suggesting that other MMPs may also be contributing to the activation of MMP-9, although in a much smaller amount. This finding is in accordance with previous reports where MMP-3 has been documented as an effective activator of proMMP-9, generating the catalytically active 82 kDa MMP-9 [35, 36]. The fact that MMP-9 activity inhibition in the zymography seems to be 100%, and the inhibition using the specific substrate assay was around 50%, may be because the latter is a more sensitive quantitative method, while zymography it is consider a qualitative assay, frequently used as semi-quantitative when optical densities of bands are measured [37, 38].

It is important to note that in almost all the experiments described in this work, measured values remain stable between 72 h and 96 h. This behavior is probably due with the fact that in culture, placental leukocytes lack the *in vivo* stimulus that induces MMP secretion, reducing progressively their production at longer times. Using the viability and functional assays, we were able to demonstrate that leukocytes are not damaged at the time experiments were done, so this is not the cause of MMP-9 and MMP-3 stabilization.

As we show here, both MMP-9 and MMP-3 are produced in larger quantities by placental leukocytes obtained from normal term labor, compared to non-labor samples. Although immunofluorescence it is not a quantitative method, it give us an insight into what is probably happening in the placental microenvironment during labor. The finding that MMP-9 and MMP-3 are increased in placental leukocytes during human labor is in accordance with previous reports showing that both enzymes increment in fetal membranes, placenta, amniotic fluid, and myometrium with the onset of term or preterm parturition, and during labor [14, 39, 40]. The fact that MMP-9 is more abundant than MMP-3 by immunofluorescence in these cells, concurs with the multiplex results, where the same tendency was observed. Our results indicate that in addition to its potential degradation activity over different fetal membrane components as collagen, proteoglycans, fibronectin, elastin and laminin [39], MMP-3 may have a key role in the onset of the extracellular matrix-degrading proteolytic cascade through proMMP-9 activation in placental leukocytes, contributing to the collagenolytic conditions observed during labor.

A limiting factor in our study is that we are just evaluating what is happening in placental leukocytes. In a broad context, we would need to consider that all gestational tissues secrete MMPs, including MMP-1, MMP-2, MMP-3, MMP-7, MMP-9 [2, 41], which can be activated each other [30, 34], integrating a network that induces the collagenolytic microenvironment observed during labor. Accordingly, our work represents one part of a bigger complex system.

In summary, in this work we show that placental leukocytes obtained from human term pregnancies are able to secrete large amounts of MMP-9, and delved into the molecular machinery required for its activation. To the best of our knowledge, we demonstrated here for the first time that MMP-3 plays a major role in such activation process in the intrauterine microenvironment. Our findings suggests that during human labor, the local activation of MMP-9 that leads to the degradation and rupture of the fetal membranes is mainly mediated by MMP-3, and that both enzymes are secreted by leukocytes circulating in the intrauterine microenvironment. These findings add temporal sense and mechanistic support to the potential role of these cells in the process of human labor.
